# Gender differences in competitiveness and fear of failure help explain why girls have lower life satisfaction than boys in gender equal countries

**DOI:** 10.3389/fpsyg.2023.1131837

**Published:** 2023-03-09

**Authors:** Kimmo Eriksson, Pontus Strimling

**Affiliations:** ^1^Institute for Futures Studies, Stockholm, Sweden; ^2^School of Education, Culture and Communication, Mälardalen University, Västerås, Sweden

**Keywords:** gender equality, gender differences, life satisfaction, competitiveness, fear of failure, equality paradox

## Abstract

Among 15-year-olds, boys tend to report higher life satisfaction than girls. Recent research has shown that this gender gap tends to be larger in more gender-egalitarian countries. We shed light on this apparent paradox by examining the mediating role of two psychological dispositions: competitiveness and fear of failure. Using data from the 2018 PISA study, we analyze the life satisfaction, competitiveness, and fear of failure of more than 400,000 15-year-old boys and girls in 63 countries with known levels of gender equality. We find that competitiveness and fear of failure together mediate more than 40 percent of the effects on life satisfaction of gender and its interaction with gender equality. Thus, interventions targeting competitiveness and fear of failure could potentially have an impact on the gender gap in life satisfaction among adolescents in gender equal countries.

## Introduction

Life satisfaction is relatively low in adolescence (Goldbeck et al., 2007; Baiocco et al., 2019; Izzo et al., 2022). Moreover, life satisfaction is typically lower among girls than boys (Inchley et al., 2016; OECD, 2017; Chen et al., 2020). Expressed differently, boys are more satisfied than girls with their life. Remarkably, it was recently demonstrated that this gender gap is wider in more gender-equal countries (Campbell et al., 2021; Guo et al., 2022). Gender equality is presumably about giving women the same privileges and opportunities as men. It is therefore paradoxical that girls are farther behind boys in life satisfaction in countries with higher gender equality. Our research question is why this paradoxical is obtained. If we can understand why life satisfaction in adolescence exhibits larger gender differences in more gender equal societies, it may suggest ways to address this undesirable gender gap.

Findings of larger gender differences in more gender equal societies have been obtained in several domains; this phenomenon has been labeled the “gender-equality paradox” (Stoet and Geary, 2020). In some domains, the gender-equality paradox has been observed among adults; examples include personality (Costa Jr et al., 2001), values (Schwartz and Rubel-Lifschitz, 2009), and preferences (Falk and Hermle, 2018). With respect to life satisfaction, however, the gender-equality paradox appears to be unique to adolescents. Among adults there is little systematic gender difference in life satisfaction, and greater gender equality is associated with higher life satisfaction among women and men alike (Batz-Barbarich et al., 2018; Audette et al., 2019).

Gender-equality paradoxes are likely to be complex phenomena. Researchers who observe the gender-equality paradox in a given domain may come up with an explanation that is specific to that domain. On the other hand, the fact that the paradox is observed across numerous different domains suggest that there may some connection between them. One possibility is that there is some truly domain-general mechanism that underlie all findings of gender-equality paradoxes. One such domain-general theory is that we will observe the gender-equality paradox whenever there are innate differences between males and females, assuming these differences are attenuated by constraints on individuality in less gender-equal societies but are allowed to develop more freely in more gender-equal countries (Falk and Hermle, 2018; Stoet and Geary, 2020). However, we do not see how this explanation could be directly applied to life satisfaction, nor how it could explain that the paradox is observed only among adolescents.

Here we shall explore another way in which findings of the gender-equality paradox for different variables may be connected. Namely, one variable could be associated with another variable at the individual level. For example, certain personality traits might lead to greater life satisfaction in adolescence. If, for some reason, the gender-equality paradox holds for those personality traits, this could be the reason why the paradox is also observed for adolescent life satisfaction.

To support this explanation, we are thus required to identify personality traits that satisfy two conditions. First, they must be able to influence life satisfaction. Second, they must themselves display the gender-equality paradox. In the present paper, we shall argue that *fear of failure* and *competitiveness* are dispositions that fit this bill.

### Fear of failure

#### How fear of failure influences life satisfaction

Psychologists have long studied the nature and consequences of dispositional fear of failure, characterized as “the capacity or propensity to experience shame upon failure” (Atkinson, 1957, p. 360). Fear of failure is thought to prompt the adoption of avoidance-based goals and strategies that have several deleterious effects (Elliot and Church, 1997). These negative implications include lowered effort, lower persistence, and poorer performance (see Elliot and Thrash, 2004). As a result, fear of failure is linked to lower life satisfaction (Elliot and Sheldon, 1997). Specifically, a negative association between fear of failure and life satisfaction is globally observed among adolescents (Govorova et al., 2020).

#### The gender-equality paradox in fear of failure

It has long been acknowledged that fear of failure is more prevalent among women than men (e.g., Macdonald and Hyde, 1980; Rothblum, 1990; Nelson et al., 2013). Little appears to be known about the development of the gender difference in fear of failure. It has been suggested that the gender differences can be attributed to sex roles (Sherman, 1988). However, it was recently demonstrated that in more gender-equal countries the gender difference in fear of failure among adolescents is *larger* (Borgonovi and Han, 2021). In other words, fear of failure exhibits the gender-equality paradox among adolescents.

The reason why the gender-equality paradox arises for fear of failure among adolescents has not been clearly established, but some ideas were sketched by Borgonovi and Han (2021). From the point of view of the present research, it only matters that the pathway they suggest does not go *via* life satisfaction, as this would ruin the logic of our hypothesis. In brief, Borgonovi and Han argued that gender equality increases not only women’s opportunities but also the societal expectations they face; for example, more gender equality tends to mean higher expectations on women than on men to manage both traditionally female and male-coded tasks (see Campbell et al., 2021). They further argued that the combination of increased opportunities and increased expectations may make women less likely to attribute failure to external constraints and more likely to internalize failure and perceive it as shameful, thus causing greater fear of failure. Note that these ideas do not involve life satisfaction.

### Competitiveness

#### How competitiveness influences life satisfaction

The literature on competitiveness has distinguished between different types of competitiveness with different consequences (Orosz et al., 2018). Our focus is on competitiveness in the broad sense of enjoying and being stimulated by competition. Competitiveness in this sense is related to personal growth, and it is associated with higher well-being (i.e., higher self-esteem and less depressive symptoms) among both boys and girls, as well as adults (Ryckman et al., 1996; Hibbard and Buhrmester, 2010). Note that research on competitiveness distinguishes the personal growth kind from the desire to win at any cost, sometimes referred to as hypercompetitiveness, which appears to have adverse social and psychological consequences (Ryckman et al., 1996; Hibbard and Buhrmester, 2010).

#### The gender-equality paradox in competitiveness

Global surveys of competitiveness among adults show a small but robust gender difference in favor of men (Van de Vliert and Janssen, 2002; Green et al., 2005). The same gender difference in competitiveness is found in economic experiments (Niederle, 2017). Although not observed among young children (Cárdenas et al., 2012), the gender difference in competitiveness is present already among adolescents (Boneva et al., 2022). Although much is unclear about why this gender difference develops, the social environment is known to play a role (Boneva et al., 2022). Importantly, the gender difference in adolescent competitiveness was recently found to be larger in countries with higher gender equality (Napp and Breda, 2022). Thus, among adolescents, competitiveness displays the gender-equality paradox.

As was the case for fear of failure, it is far from settled why the gender-equality paradox arises in the case of competitiveness. We briefly summarize the ideas offered by Napp and Breda (2022). They argue that gender-equal societies tend to value self-realization and self-expression. These societal values are thought to create a need in individuals to express their social identities, which may lead them to fall back on and internalize essentialist gender norms (e.g., Charles et al., 2014). Specifically, they may adopt more gendered ideas about talent and, for this reason, girls in more gender-equal societies would be less willing to enter competitive situations. From the point of view of the present research, it only matters that these ideas do not involve low life satisfaction as an antecedent of fear of failure.

### Hypothesis and aim of study

**Figure 1:**
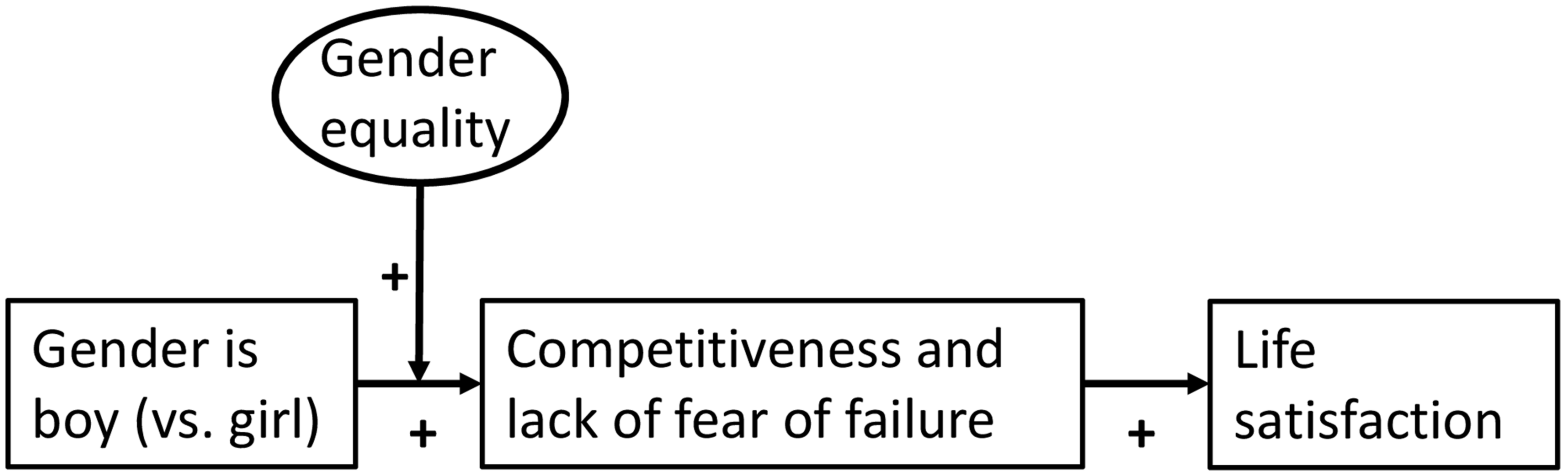
A mediation model. We hypothesize that the gender-equality paradox for adolescent life satisfaction is mediated by competitiveness and lack of fear of failure. These dispositions display the gender-equality paradox too; that is, they are higher among boys than girls and especially in more gender-equal societies. In the diagram, boxes represent individual-level variables while the oval represents a society-level variable.

As detailed above, prior research supports the existence of the links in Figure 1. An implication of this diagram is that fear of failure and competitiveness should, at least partially, mediate the effects on adolescent life satisfaction of gender and its interaction with gender equality. The aim of our study is to test this hypothesis using cross-cultural survey data.

## Materials and methods

Our main data source is the 2018 wave of PISA, the OECD’s Program for International Student Assessment, which targets 15-year-olds. PISA includes achievement tests as well as extensive questionnaires. The 2018 student questionnaire included measures of life satisfaction, fear of failure, and competitiveness. The same dataset was used in several of the studies reviewed above (Govorova et al., 2020; Borgonovi and Han, 2021; Campbell et al., 2021; Kuhn et al., 2021; Napp and Breda, 2022).

### Countries

PISA covers a great number of countries, including a few country-like entities like Hong Kong. We use data on every country in the 2018 PISA for which there is also data on gender equality from the World Values Survey, totaling 63 countries. Supplementary Table 1 lists the countries in our study and the key measures aggregated by country and gender.

### Participants

We use data on all 2018 PISA participants of known gender who completed measures of life satisfaction, fear of failure, and competitiveness, totaling 408,254 students (50.9% girls and 49.1% boys). The sample size per country ranged between 2,968 and 32,945. The percentage of girls per country ranged between 47.9% and 54.7%.

### PISA measures

#### Life satisfaction

The PISA measure of life satisfaction consists of a single item: “Overall, how satisfied are you with your life as a whole these days?.” Responses were given on a scale from 0, representing not at all satisfied, to 10, representing completely satisfied.

#### Fear of failure

To cover several facets of fear of failure (Conroy, 2001), PISA includes three items: “When I am failing, I worry about what others think of me,” “When I am failing, I am afraid that I might not have enough talent,” and “When I am failing, this makes me doubt my plans for the future.” Responses were given on a four-point Likert scale ranging from “Strongly disagree” (coded 1) to “Strongly agree” (coded 4). The items have adequate internal consistency (Cronbach’s alpha = 0.81 in the average country, see Table 16.29 in the PISA 2018 Technical Report^1^). The average of the three items serves as our measure of the participant’s fear of failure.

#### Competitiveness

To cover several aspects of competitiveness (Orosz et al., 2018), PISA includes three items: “I enjoy working in situations involving competition with others,” “It is important for me to perform better than other people on a task,” and “I try harder when I’m in competition with other people.” Responses were given on the same four-point Likert scale, as for fear of failure. The items have adequate internal consistency (Cronbach’s alpha = 0.77 in the average country, see Table 16.29 in the PISA 2018 Technical Report). We use the average of the three items to measure the participant’s competitiveness.^2^

### Gender equality measures

There are several ways to operationalize gender equality. The World Values Survey provides a measure of normative gender equality, that is, cultural values with respect to treating men and women equally or differently (Welzel, 2013). An alternative is the Global Gender Gap Index (GGGI), which estimates gender equality in outcomes in the form of national gaps between men and women with respect to economic participation and wages, educational attainment, political empowerment, and health and survival. As different levels of structural gender equality may result in the same outcome, the GGGI may be less suitable to represent sex equality as a causal factor (Richardson et al., 2020). Consistent with this view, several prior studies predicting gender differences by gender equality have found stronger results for normative gender equality than for gender equality in outcomes (Eriksson et al., 2020, 2022). In the present study we include both measures of gender equality as detailed below.

#### WVS: Normative gender equality

The World Values Survey (WVS; Haerpfer et al., 2021) and the European Values Study (2021) both include the EQUALITY index consisting of three items (“When jobs are scarce, men should have more right to a job than women,” “On the whole, men make better political leaders than women do,” “University is more important for a boy than for a girl”). Responses on the three items are averaged, yielding a measure between 0 and 1 for each participant. We take the country mean for the most recent wave in which the country participated, multiplied by 100 to obtain country measures on a convenient scale between 0 and 100. For a few countries there was data on the underlying jobs-related item but not on the full EQUALITY index. As the jobs item is extremely strongly correlated with the full index, *r* = 0.97, we used the country means for the jobs item in a linear prediction to estimate EQUALITY index scores for these countries, following Eriksson et al. (2022). The resulting scores are reported in Supplementary Table 1.

#### GGGI: Gender equality in outcomes

GGGI data for 59 countries (but not for Taiwan, Hong Kong, Macao, and Kosovo) are obtained from the Global Gender Gap Report for 2018 (World Economic Forum, 2018). See Supplementary Table 1.

### GDP *per capita* and Gini

The original study of Campbell et al. (2021) found that the relation between gender differences in life satisfaction and gender equality holds even when controlling for GDP *per capita* (a measure of economic development) and Gini (a measure of economic inequality). In order to examine whether this holds also for competitiveness and fear of failure, we downloaded data on GDP *per capita* for 2018 and Gini from the World Bank.^3^ Data on GDP *per capita* were available for 62 countries in our study. Gini data were available for 47 countries.

### Analysis plan

We begin by calculating country-level gender differences in life satisfaction, competitiveness, and fear of failure, to establish that boys typically score higher on life satisfaction and competitiveness, and that girls typically score higher on fear of failure.

Next, we examine how these country-level gender differences correlate with gender equality, to establish that more gender-equal countries have larger gender differences in life satisfaction, fear of failure, and competitiveness, both with and without controlling for GDP *per capita* or Gini. (We use Pearson correlation, which is equivalent to the standardized coefficient of a simple linear regression and thus assumes normality of residuals and homoskedasticity. The validity of these assumptions was confirmed by examination of normal P–P plots and scatter plots of residuals against predicted values^4^; these analyses are not included here.)

We then turn to individual-level analyses of how life satisfaction correlates with competitiveness and fear of failure, to establish that life satisfaction is positively correlated with competitiveness and negatively correlated with fear of failure.

The last step is a mixed-level analysis aimed at examining the extent to which competitiveness and fear of failure mediate the effects on life satisfaction of gender and its interaction with the level of gender equality of the country. The mediation analysis consists of estimation of two mixed-level models of life satisfaction. The first model includes only gender, gender equality, and the interaction term. The second model additionally includes competitiveness and fear of failure. Mediation is quantified by the extent to which inclusion of competitiveness and fear of failure reduce the main effect of gender and the interaction between gender and gender equality.

## Results

### Gender differences

Table 1 reports descriptive statistics for life satisfaction, fear of failure, and competitiveness aggregated to the country level. Note that life satisfaction is measured on a scale from 0 to 10, so the mean value of 7.2 lies in the mid-upper range of the scale. Fear of failure and competitiveness are measured on a scale from 1 to 4, so the mean values of 2.5 and 2.8 lie in the middle of the scale. Table 1 also reports the mean gender difference, showing that boys score higher on life satisfaction and competitiveness while girls score higher on fear of failure. The average effect size of gender is largest for fear of failure.

**Table 1:** Descriptive statistics of country-level variables.

Variable	Mean	SD	GD	*d*	*t*
Life Satisfaction	7.2 (0.6)	2.5 (0.3)	0.43 (0.33)	0.18 (0.13)	6.9 (5.3)
Competitiveness	2.8 (0.2)	0.7 (0.1)	0.12 (0.12)	0.18 (0.17)	6.7 (7.8)
Fear of Failure	2.5 (0.2)	0.8 (0.1)	−0.21 (0.14)	−0.27 (0.17)	−10.7 (7.1)

Based on *N* = 63 countries. Entries are mean values with standard deviations in parentheses. SD: within-country standard deviation; GD: gender difference calculated as boys’ mean value minus girls’ mean value; *d*: gender effect size calculated as GD divided by SD; t: GD divided by the standard error of GD.

### The relation between gender differences and gender equality

Table 2 reports Pearson correlations between the above gender differences and measures of gender equality. Replicating prior findings, more gender-equal countries have larger gender differences in life satisfaction, fear of failure, and competitiveness. Correlations with gender differences are consistently stronger for the WVS measure of gender equality than for the GGGI measure. Thus, in the remaining analyses, we focus exclusively on the WVS measure.

**Table 2:** Pearson and partial correlations between gender differences and measures of gender equality.

Gender difference	GGGI (*N* = 59)	WVS (*N* = 63)	WVS controlling for GDP/cap (*N* = 62)	WVS controlling for Gini (*N* = 47)
Life Satisfaction	0.45	0.65	0.63	0.64
Competitiveness	0.57	0.76	0.74	0.68
Fear of Failure	−0.65	−0.65	−0.62	−0.53

The last two columns report partial correlations.

The last two columns of Table 2 show that correlations with the WVS measure of gender equality are essentially unchanged when we control for economic development or economic inequality. We conclude that these variables are not important confounders of the relation between gender differences and gender equality.

**Figure 2:**
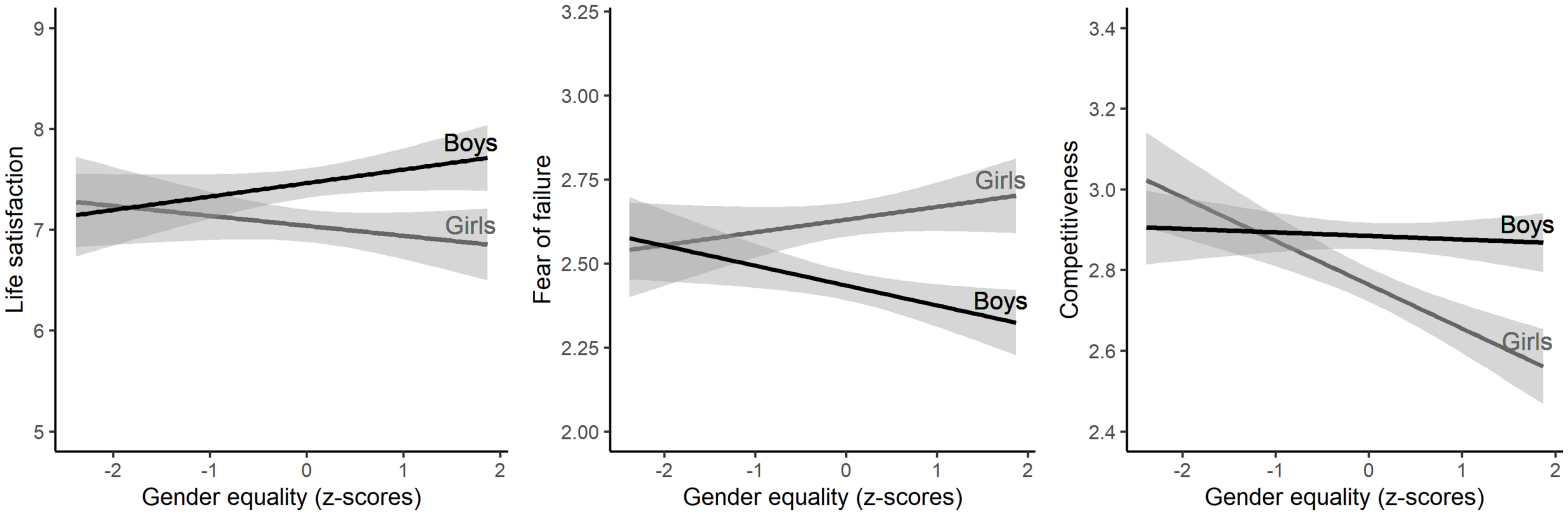
Estimated linear relations between the WVS measure of gender equality of a country and boys’ and girls’ average life satisfaction (left), average fear of failure (middle), and average competitiveness (right). Shaded areas indicate 95% confidence intervals of the regression lines.

To show the relation between gender equality and gender differences in more detail, Figure 2 illustrates how the mean scores of boys and the mean scores of girls varied with the gender equality of the country. The association between life satisfaction and gender equality is positive for boys and negative for girls. The association between fear of failure and gender equality is negative for boys and positive for girls. The association between competitiveness and gender equality is almost null for boys and very negative for girls.

### Individual-level links between psychological dispositions and life satisfaction

**Figure 3:**
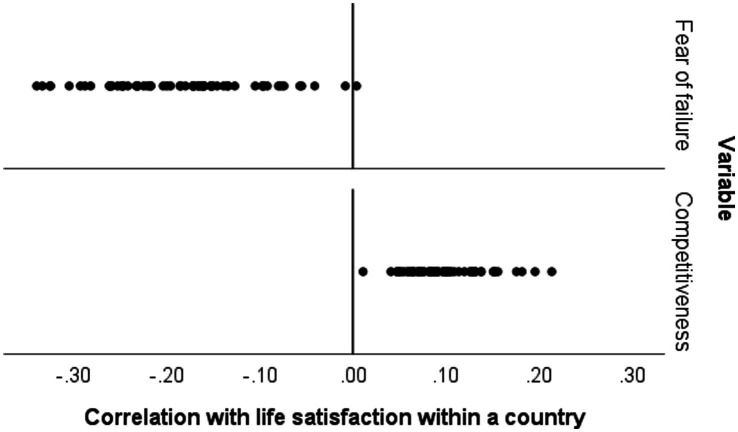
Within-country correlations, in 63 countries, between life satisfaction and fear of failure (top) and between life satisfaction and competitiveness (bottom).

Consistent with prior research, life satisfaction is positively correlated with competitiveness in every country and negatively correlated with fear of failure in all countries but one, see Figure 3. (Note that these correlations have some sampling error, so the exceptional case may well be spurious).

### Individual-level links between gender and psychological dispositions

We perform mixed-level analyses to estimate the individual-level effects on psychological dispositions of gender and its interaction with gender equality. The models include fixed effects of the individual’s gender (1 for boy, 0 for girl), the country’s level of gender equality in the WVS (z-scored, that is, standardized to have mean zero and standard deviation 1), and their cross-level interaction, as well as random intercept and random slope for gender at the country level. Fear of failure and competitiveness were analyzed in separate models. Results are reported in Table 3. The interpretation of the key results for fear of failure is as follows: In a country with an average level of gender equality, the average fear of failure of boys is estimated to be 0.21 points lower than that of girls; this negative gender difference is larger in more gender-equal countries, and the slope is estimated to be −0.10 points per standard deviation of gender equality. Competitiveness, on the other hand, is on average 0.12 points higher for boys than girls in a country with an average level of gender equality, and this positive gender difference is larger in more gender-equal countries, at a slope of 0.10 points per standard deviation of gender equality.

**Table 3:** Results of mixed-level analyses of fear of failure and competitiveness.

Regressor	DV: Fear of failure	DV: Competitiveness
Intercept	2.64 [2.59, 2.69]	2.76 [2.72, 2.80]
Gender	−0.21 [−0.23, -0.18]	0.12 [0.10, 0.14]
Equality	0.04 [−0.02, 0.09]	−0.11 [−0.15, −0.06]
Gender × Equality	−0.10 [−0.12, -0.07]	0.10 [0.08, 0.12]
Residual variance:
Individual	0.58	0.48
Country, intercept	0.04	0.03
Country, gender slope	0.01	0.01
AIC	939,314	860,277
BIC	939,353	860,310
Pseudo *R*^2^, marginal	0.020	0.019
Pseudo *R*^2^, conditional	0.096	0.076

Based on 408,254 students in 63 countries. Entries are unstandardized coefficients with 95% confidence intervals. Gender is a dummy variable coded 1 for boy, 0 for girl. Equality refers to the z-scored WVS measure of gender equality of countries.

### Mediation analysis

Our hypothesis is that fear of failure and competitiveness mediate the effects on life satisfaction of gender and its interaction with gender equality. To examine this hypothesis, we need to perform two analyses, in which we first estimate these effects on life satisfaction and then estimate the extent to which the effects are accounted for when the mediators (fear of failure and competitiveness) are included in the analysis, the *proportion mediated* (MacKinnon et al., 2007). The results are reported in Table 4. In a country with an average level of gender equality, the average life satisfaction of boys is estimated to be 0.42 points higher than that of girls (Model 1), and this effect of gender is decreased to 0.25 when fear of failure and competitiveness is included (Model 2). The proportion of the effect of gender that was mediated by the psychological dispositions was 40.4 percent, with a 95% bootstrap confidence interval between 40.3 and 40.4 percent obtained from analyses of 1,000 bootstrap samples.

**Table 4:** Results of mixed-level analyses of life satisfaction.

Regressor	Model 1	Model 2
Intercept	7.04 [6.88, 7.20]	7.57 [7.43, 7.71]
Gender	0.42 [0.36, 0.49]	0.25 [0.20, 0.31]
Equality	−0.10 [−0.27, 0.07]	−0.03 [−0.19, 0.12]
Gender × Equality	0.23 [0.16, 0.30]	0.13 [0.07, 0.20]
Fear of failure		−0.61 [−0.62, −0.60]
Competitiveness		0.39 [0.37, 0.40]
Residual variance:		
Individual	6.14	5.89
Country, intercept	0.40	0.31
Country, gender slope	0.06	0.05
AIC	1,900,307	1,882,858
BIC	1,900,340	1,882,891
Pseudo *R*^2^, marginal	0.009	0.050
Pseudo *R*^2^, conditional	0.073	0.101

Based on 408,254 students in 63 countries. Entries are unstandardized coefficients with 95% confidence intervals. Gender is a dummy variable coded 1 for boy, 0 for girl. Equality refers to the z-scored WVS measure of gender equality of countries.

Similarly, the interaction between gender and gender equality means that the effect of gender on life satisfaction is 0.23 points larger when gender equality is one standard deviation higher of gender equality (Model 1), and this interaction effect is decreased to 0.13 when fear of failure and competitiveness is included (Model 2). The proportion of the interaction effect that was mediated by the psychological dispositions was 41.8 percent, 95% bootstrap CI [41.7, 41.9]. Thus, while full mediation was not achieved, fear of failure and competitiveness were able to account for almost half of the gender-equality paradox.

For completeness, we also performed the same analysis replacing the WVS measure of gender equality with the GGGI and focusing on the 59 countries for which GGGI data are available (Supplementary Table 1). Mediation was even a bit stronger in this analysis; inclusion of fear of failure and competitiveness decreased the interaction between gender and GGGI by 55 percent.

## Discussion

In this paper, we have addressed the gender-equality paradox in adolescent life satisfaction. How can it be that the difference in life satisfaction between adolescent boys and girls is larger in more gender-equal countries? We proposed a novel kind of explanation: that the gender-equality paradox for life satisfaction can be pushed back to similar paradoxes for psychological dispositions that affect life satisfaction. Specifically, we proposed that fear of failure and competitiveness may play this mediating role.

We examined the life satisfaction, fear of failure, and competitiveness of 15-year-olds in 63 countries around the world. Replicating prior findings, we observed a male advantage in all three measures. In other words, boys are more satisfied with their life than girls are (Inchley et al., 2016), boys experience less fear of failure than girls do (Borgonovi and Han, 2021), and boys are more competitive than girls (Boneva et al., 2022). Moreover, in more gender-equal countries, we observe wider gender gaps in life satisfaction (Campbell et al., 2021), fear of failure (Borgonovi and Han, 2021), and competitiveness (Napp and Breda, 2022). Our study appears to be the first to examine these phenomena simultaneously.

We found the correlation between gender gaps and gender equality to be stronger for psychological dispositions (fear of failure and competitiveness) than for life satisfaction. This finding is in keeping with our hypothesis that the pathway by which gender and gender equality affect life satisfaction goes *via* psychological dispositions (Figure 2). More evidence for this hypothesis was obtained in a mediation analysis, which showed that fear of failure and competitiveness could account for 40 percent of the effects on life satisfaction of gender and its interaction with gender equality.

This work has both theoretical and practical implications. From a theoretical point of view, we have demonstrated a way to connect different instances of the gender-equality paradox. If X and Y are two individual-level variables and X influences Y, then the gender-equality paradox may hold for Y simply because it holds for X. In the case studied in the present research, X were certain dispositions and Y was life satisfaction. The same logic could potentially be used to connect other of the numerous instances of the gender-equality paradox. For example, it could be that the gender-equality paradox in personality (Costa et al., 2001) underlies several other instances of the paradox for variables that are influenced by personality. This is a topic for future research.

From a practical point of view, our findings describe both a problem and a potential solution. The problem is that girls’ life satisfaction is especially low in gender-equal societies. The potential solution is that interventions that target girls’ low competitiveness and high fear of failure in these societies could also be a way of achieving greater life satisfaction. How to conduct such interventions is beyond the scope of this study. The literature on interventions includes studies on both competitiveness (Boneva et al., 2022) and fear of failure (Stamps, 1973; Martin and Marsh, 2003), which may be a starting point.

An important limitation of the current study is that it relies on cross-sectional data, which do not provide information on causal directions. Thus, from the data we cannot exclude the possibility that the causal direction goes in reverse, that is, that young people’s level of life satisfaction may influence their competitiveness and fear of failure. Intervention studies may also provide evidence for our working assumption about the causal direction.

Another limitation is that life satisfaction was measured by a single item. Such measures are sensitive to individual differences in response style. Response style could similarly bias the measure of competitiveness, because although it is measured using several items, they are all coded in the same direction. The same goes for fear of failure. However, it is unlikely that our findings are driven by response style, as competitiveness and lack of fear of failure are coded in different directions yet give similar results.

## Data availability statement

Publicly available datasets were analyzed in this study. This data can be found here: The data on gender value equality, presented in Supplementary Table 1, were derived from the joint WVS/EVS database available in the public domain (https://www.worldvaluessurvey.org/WVSEVStrend.jsp). The data from 2018 PISA are available in the public domain (https://www.oecd.org/pisa/).

## Ethics statement

Ethical review and approval was not required for the study on human participants in accordance with the local legislation and institutional requirements. Written informed consent from the participants’ legal guardian/next of kin was not required to participate in this study in accordance with the national legislation and the institutional requirements.

## Author contributions

KE and PS conceived of the study. KE performed the analyses and wrote the paper. PS provided critical revisions. All authors contributed to the article and approved the submitted version.

## Funding

This research was funded by the Knut and Alice Wallenberg Foundation (grant no. 2017.0257) and the Swedish Research Council (grant no. 2014–2008).

## Conflict of interest

The authors declare that the research was conducted in the absence of any commercial or financial relationships that could be construed as a potential conflict of interest.

## Publisher’s note

All claims expressed in this article are solely those of the authors and do not necessarily represent those of their affiliated organizations, or those of the publisher, the editors and the reviewers. Any product that may be evaluated in this article, or claim that may be made by its manufacturer, is not guaranteed or endorsed by the publisher.

## Supplementary material

The Supplementary material for this article can be found online at: https://www.frontiersin.org/articles/10.3389/fpsyg.2023.1131837/full#supplementary-material

## Footnotes

^1^ https://www.oecd.org/pisa/data/pisa2018technicalreport

^2^We also subjected the six items on fear of failure and competitiveness to an exploratory factor analysis in SPSS (principal axis factoring, oblimin rotation with Kaiser normalization). The expected two-factor structure was observed, and the two factors together explain more than 70 percent of the total variance.

^3^ https://data.worldbank.org/

^4^See statisticssolutions.com/testing-assumptions-of-linear-regression-in-spss
