# Supplementary material for: Gender differences in competitiveness and fear of failure help explain why girls have lower life satisfaction than boys in gender equal countries
Source: Front Psychol. 2023 Mar 9;14:1131837. doi: 10.3389/fpsyg.2023.1131837 (PMC10034386; doi:10.3389/fpsyg.2023.1131837)
Supplement: Supplementary file 1 [file Table_1.DOCX]

**Supplementary Table 1**. Values of variables per country.

| COUNTRY | N boys | N girls | M LS boys | M LS girls | M Co boys | M Co girls | M FoF boys | M FoF girls | WVS | GGGI |
| --- | --- | --- | --- | --- | --- | --- | --- | --- | --- | --- |
| Albania | 2825 | 2978 | 8.66 | 8.56 | 0.59 | 0.70 | -0.26 | -0.23 | 77 | 0.73 |
| Argentina | 4693 | 5307 | 7.59 | 7.07 | 0.03 | -0.27 | -0.22 | -0.01 | 74 | 0.73 |
| Austria | 3131 | 3140 | 7.58 | 6.84 | 0.11 | -0.20 | -0.43 | -0.11 | 79 | 0.72 |
| Belarus | 2899 | 2666 | 8.22 | 7.91 | 0.04 | -0.08 | -0.29 | 0.04 | 60 | 0.75 |
| Bosnia and Herzegovina | 2802 | 2811 | 8.09 | 7.54 | 0.16 | -0.09 | -0.44 | -0.31 | 64 | 0.71 |
| Brazil | 3833 | 4287 | 7.36 | 6.75 | 0.07 | -0.22 | -0.10 | 0.16 | 72 | 0.68 |
| Bulgaria | 2011 | 2055 | 7.42 | 7.00 | 0.03 | -0.04 | -0.22 | -0.03 | 63 | 0.76 |
| Chile | 3258 | 3333 | 7.40 | 6.87 | 0.21 | -0.01 | -0.02 | 0.23 | 59 | 0.72 |
| Chinese Taipei | 3468 | 3506 | 6.77 | 6.29 | 0.34 | 0.30 | 0.53 | 0.79 | 63 |  |
| Colombia | 3083 | 3286 | 7.79 | 7.36 | 0.26 | 0.03 | -0.28 | -0.12 | 67 | 0.73 |
| Croatia | 3034 | 3180 | 8.11 | 7.23 | 0.08 | -0.28 | -0.37 | -0.08 | 76 | 0.71 |
| Czech Rep | 3188 | 3235 | 7.23 | 6.62 | 0.05 | -0.22 | -0.23 | 0.17 | 66 | 0.69 |
| Dominican Rep | 1475 | 1659 | 8.30 | 7.83 | 0.17 | 0.00 | -0.06 | 0.02 | 71 | 0.70 |
| Estonia | 2501 | 2551 | 7.52 | 6.85 | 0.01 | -0.23 | -0.39 | 0.06 | 73 | 0.73 |
| Finland | 2623 | 2634 | 8.03 | 7.21 | 0.11 | -0.16 | -0.46 | 0.08 | 84 | 0.82 |
| France | 2825 | 2813 | 7.38 | 6.91 | 0.07 | -0.37 | -0.16 | 0.31 | 86 | 0.78 |
| Georgia | 2109 | 2280 | 7.66 | 7.50 | 0.03 | 0.12 | -0.39 | -0.27 | 53 | 0.68 |
| Germany | 2143 | 1971 | 7.37 | 6.66 | 0.10 | -0.23 | -0.58 | -0.16 | 83 | 0.78 |
| Greece | 2903 | 2998 | 7.21 | 6.75 | 0.12 | -0.15 | -0.26 | 0.08 | 65 | 0.70 |
| Hong Kong | 2829 | 2782 | 6.32 | 6.22 | 0.14 | 0.13 | 0.26 | 0.55 | 61 |  |
| Hungary | 2357 | 2478 | 7.50 | 6.73 | 0.24 | -0.07 | -0.35 | 0.14 | 68 | 0.67 |
| Iceland | 1432 | 1536 | 7.79 | 6.93 | 0.10 | -0.16 | -0.31 | 0.30 | 89 | 0.86 |
| Indonesia | 5314 | 5768 | 7.47 | 7.24 | 0.15 | 0.20 | -0.21 | -0.10 | 35 | 0.69 |
| Ireland | 2661 | 2669 | 7.01 | 6.44 | 0.35 | -0.02 | -0.04 | 0.45 | 75 | 0.80 |
| Italy | 5351 | 5132 | 7.33 | 6.65 | 0.24 | -0.04 | -0.20 | 0.15 | 72 | 0.71 |
| Japan | 2898 | 3050 | 6.20 | 6.20 | -0.20 | -0.19 | 0.25 | 0.51 | 62 | 0.66 |
| Jordan | 3703 | 4374 | 6.59 | 7.10 | 0.39 | 0.60 | -0.19 | -0.21 | 37 | 0.61 |
| Kazakhstan | 8929 | 8880 | 8.60 | 8.39 | -0.46 | -0.41 | -0.41 | -0.20 | 48 | 0.71 |
| Kosovo | 2175 | 2200 | 8.44 | 8.20 | 0.28 | 0.18 | -0.14 | -0.01 | 70 |  |
| Latvia | 2400 | 2543 | 7.42 | 6.90 | 0.01 | -0.11 | -0.31 | 0.09 | 74 | 0.76 |
| Lebanon | 1881 | 2181 | 6.61 | 6.76 | 0.31 | 0.32 | -0.20 | -0.16 | 54 | 0.60 |
| Lithuania | 3163 | 3174 | 7.85 | 7.33 | 0.09 | -0.01 | -0.29 | 0.12 | 63 | 0.75 |
| Luxembourg | 2386 | 2380 | 7.35 | 6.75 | 0.00 | -0.31 | -0.36 | 0.09 | 77 | 0.71 |
| Macao | 1893 | 1840 | 6.22 | 5.93 | 0.16 | 0.13 | 0.30 | 0.59 | 60 |  |
| Malaysia | 2851 | 3009 | 7.10 | 7.02 | 0.41 | 0.47 | 0.24 | 0.46 | 47 | 0.68 |
| Malta | 1482 | 1535 | 6.89 | 6.23 | 0.46 | 0.20 | 0.06 | 0.44 | 68 | 0.69 |
| Mexico | 2651 | 2987 | 8.29 | 7.96 | 0.27 | 0.08 | 0.03 | 0.09 | 67 | 0.72 |
| Moldova | 2548 | 2529 | 7.62 | 7.79 | 0.18 | 0.10 | -0.17 | 0.10 | 54 | 0.73 |
| Montenegro | 2863 | 2983 | 7.96 | 7.41 | 0.21 | -0.11 | -0.43 | -0.42 | 59 | 0.71 |
| Morocco | 2286 | 2166 | 7.16 | 6.81 | 0.28 | 0.37 | -0.22 | -0.07 | 46 | 0.61 |
| Netherlands | 1857 | 1882 | 7.83 | 7.17 | 0.04 | -0.25 | -0.65 | -0.14 | 82 | 0.75 |
| North Macedonia | 2521 | 2348 | 8.26 | 8.12 | 0.50 | 0.44 | -0.18 | -0.11 | 65 | 0.71 |
| Peru | 2300 | 2155 | 7.47 | 7.06 | 0.33 | 0.23 | -0.23 | -0.17 | 69 | 0.72 |
| Philippines | 2848 | 3289 | 7.02 | 7.46 | 0.12 | 0.05 | 0.09 | 0.25 | 39 | 0.80 |
| Poland | 2602 | 2768 | 7.16 | 6.35 | 0.06 | -0.15 | -0.19 | 0.22 | 69 | 0.73 |
| Portugal | 2711 | 2728 | 7.42 | 6.90 | 0.20 | -0.32 | -0.21 | 0.19 | 72 | 0.73 |
| Qatar | 5165 | 6253 | 7.00 | 6.74 | 0.30 | 0.38 | -0.06 | -0.03 | 35 | 0.63 |
| Romania | 2461 | 2369 | 7.99 | 7.79 | 0.36 | 0.27 | -0.40 | -0.14 | 59 | 0.71 |
| Russian Federation | 3291 | 3545 | 7.52 | 7.00 | 0.02 | -0.11 | -0.29 | -0.03 | 51 | 0.70 |
| Saudi Arabia | 2721 | 2843 | 7.80 | 8.03 | 0.36 | 0.44 | -0.20 | -0.45 | 28 | 0.59 |
| Serbia | 2633 | 2829 | 7.81 | 7.38 | -0.06 | -0.45 | -0.39 | -0.24 | 68 | 0.73 |
| Slovak Republic | 2600 | 2717 | 7.62 | 6.85 | 0.00 | -0.20 | -0.14 | 0.17 | 54 | 0.69 |
| Slovenia | 3038 | 2808 | 7.37 | 6.24 | 0.02 | -0.33 | -0.24 | 0.22 | 76 | 0.78 |
| South Korea | 3397 | 3153 | 6.98 | 5.99 | 0.09 | -0.10 | 0.03 | 0.37 | 46 | 0.66 |
| Spain | 16353 | 16592 | 7.55 | 7.11 | 0.21 | -0.13 | -0.24 | 0.03 | 83 | 0.75 |
| Sweden | 2434 | 2568 | 7.47 | 6.58 | 0.12 | -0.24 | -0.28 | 0.27 | 93 | 0.82 |
| Switzerland | 2642 | 2499 | 7.70 | 7.05 | -0.05 | -0.40 | -0.41 | -0.08 | 81 | 0.76 |
| Thailand | 3748 | 4479 | 7.66 | 7.49 | 0.06 | 0.00 | 0.11 | 0.27 | 56 | 0.70 |
| Turkey | 3266 | 3245 | 5.72 | 5.50 | 0.35 | 0.31 | 0.03 | 0.21 | 49 | 0.63 |
| Ukraine | 2991 | 2790 | 8.03 | 8.02 | -0.21 | -0.36 | -0.34 | -0.08 | 57 | 0.71 |
| United Kingdom | 6024 | 6476 | 6.70 | 5.94 | 0.35 | -0.07 | -0.02 | 0.57 | 81 | 0.77 |
| United States | 2291 | 2266 | 7.04 | 6.51 | 0.41 | 0.09 | -0.06 | 0.35 | 78 | 0.72 |
| Uruguay | 1821 | 2194 | 7.96 | 7.25 | 0.08 | -0.35 | -0.15 | -0.02 | 75 | 0.72 |

Note. M = mean value. LS = life satisfaction. Co = competitiveness. FoF = fear of failure. WVS = the WVS measure of gender equality. GGGI = the GGGI measure of gender equality.
