# Supplementary material for: Gender differences in competitiveness and fear of failure help explain why girls have lower life satisfaction than boys in gender equal countries
Source: Front Psychol. 2023 Mar 9;14:1131837. doi: 10.3389/fpsyg.2023.1131837 (PMC10034386; doi:10.3389/fpsyg.2023.1131837)
Supplement: Supplementary file 2 [file Table_2.DOCX]

**Supplementary Table 2.** Results of mixed-level analyses of life satisfaction using the GGGI measure of gender equality.

| Regressor | Model 1 | Model 2 |
| --- | --- | --- |
| Intercept | 7.06 [6.90,7.22] | 7.60 [7.46,7.75] |
| Gender | 0.44 [0.36,0.52] | 0.26 [0.20,0.33] |
| Equality | 0.02 [-0.13,0.17] | 0.08 [-0.05,0.21] |
| Gender ⨉ Equality | 0.14 [0.07,0.22] | 0.06 [0.01,0.12] |
| Fear of failure |  | -0.61 [-0.62,-0.60] |
| Competitiveness |  | 0.38 [0.37,0.39] |
| Residual variance: |  |  |
| individual | 6.18 | 5.92 |
| country, intercept | 0.37 | 0.28 |
| country, gender slope | 0.09 | 0.06 |
| AIC | 1,806,527 | 1,789,809 |
| BIC | 1,806,559 | 1,789,842 |
| Pseudo R^2^, marginal | 0.009 | 0.051 |
| Pseudo R^2^, conditional | 0.071 | 0.097 |

Note. Based on 387,561 students in 59 countries. Entries are unstandardized coefficients with 95% confidence intervals. Gender is a dummy variable coded 1 for boy, 0 for girl. Equality refers to the z-scored GGGI measure of gender equality of countries.
